# Putting behaviors into context for vector-borne diseases: Examining behaviors that may reduce exposure to disease vectors

**DOI:** 10.1371/journal.pntd.0013365

**Published:** 2025-08-12

**Authors:** Raissa Nogueira de Brito, Susan Tanner, Julie Velásquez Runk, Nicole L. Gottdenker, Adelys Reina, Ayesha Rodríguez, Chystrie Rigg, Daniel Mendieta, John P. Schmidt, Juliana Hoyos, Kadir González, Kimberly Archbold, Richard J. Hall, Tania Gómez, Vanessa Pineda, Vanessa Vásquez, Jose E. Calzada, Azael Saldaña

**Affiliations:** 1 Department of Anthropology, University of Georgia, Athens, Georgia, United States of America; 2 Department of Pathology, College of Veterinary Medicine, University of Georgia, Athens, Georgia, United States of America; 3 Environment and Sustainability Studies Program, Wake Forest University, Winston-Salem, North Carolina, United States of America; 4 Smithsonian Tropical Research Institute, Panama City, Republic of Panama; 5 Departamento de Investigación en Parasitología, Instituto Conmemorativo Gorgas de Estudios de Salud (ICGES), Panama City, Republic of Panama; 6 Odum School of Ecology, University of Georgia, Athens, Georgia, United States of America; 7 Department of Infectious Diseases, College of Veterinary Medicine, University of Georgia, Athens, Georgia, United States of America; 8 Facultad de Medicina, Centro de Investigación y Diagnóstico de Enfermedades Parasitarias (CIDEP), Universidad de Panamá, Panama City, Republic of Panama; Egerton University, KENYA

## Abstract

Understanding why people adopt or ignore vector-borne disease (VBD) preventive measures is key to better risk assessment and control. However, little is known about why some households adopt preventive practices while others do not, which is particularly striking in this era of One Health’s attention to human, environmental, and animal health. We examined what household factors are linked to practices that may reduce exposure to disease vectors, with a focus on Chagas disease (CD) and American Cutaneous Leishmaniasis (ACL) vectors. We surveyed 204 households in 12 rural communities in Coclé Province, Republic of Panama, between March 2022 and December 2023. We used logistic regression models to explore the association between household contextual elements, such as respondents’ sex, wealth (consumer goods and agricultural), knowledge about ACL and CD, feelings about insects, and perceptions of control over health outcomes, and personal (use of repellents, and hand protection before touching a CD vector) and property-based behaviors (use of insecticides in the house, use of windows/doors screens, and cleaning peridomestic debris) that may reduce exposure to disease vectors. We found mixed associations between protective behaviors and the contextual household elements*.* Participants who reported feeling bothered by insects were more likely to use repellents (Odds Ratio [OR]: 2.97 95% Confidence Interval [95%CI]: 1.47-6.20), whereas those who reported being in control of their health were less likely to use protection before touching a CD vector (OR = 0.8, CI: 0.67-0.95). Window/door screens were associated with more household consumer goods wealth (OR: 1.44; CI: 1.23-1.71), while households with a history of ACL cases were more likely to have peridomestic debris accumulation (OR: 2.33; CI: 1.17-4.75). Additionally, householders bothered by insects were less likely to have peridomestic debris (OR: 0.42; CI: 0.20-0.84), as well as those who believe health outcomes happen by chance (OR: 0.89; CI: 0.82-0.98). Our findings emphasize the importance of considering a more comprehensive household background and attention to cultural values to foster context-sensitive strategies for vector-borne pathogen prevention and control.

## Author summary

Preventing vector-borne diseases, such as American Cutaneous Leishmaniasis (ACL) and Chagas disease (CD), requires understanding why some households adopt protective measures while others do not. In this study, we surveyed 204 households across 12 rural communities in Coclé Province, Republic of Panama, to explore how factors like wealth, disease knowledge, people’s feelings towards insects, and perception of their health outcomes influence preventive behaviors for disease vectors. Our findings suggest that individuals more sensitive to insect presence are more likely to use protective measures like repellents, while those with a stronger sense of control over their health may be less inclined to take precautions to touch a CD vector. Additionally, households with a greater number of consumer goods and appliances are more likely to implement structural barriers in their houses to prevent contact with disease vectors, whereas a history of ACL cases in the household is linked to peridomestic environmental conditions that could influence insect presence. These results highlight the complex relationship between individuals’ feelings, perceptions, household conditions, and vector-borne disease prevention behaviors. Disease control strategies that consider local knowledge, cultural values, and more comprehensive household contexts can improve vector control efforts and reduce the burden of vector-borne diseases.

## Introduction

Vector-borne pathogens transmission requires effective preventive measures to mitigate their public health impact [1]. Preventive measures involve personal and property-based strategies to deter vectors from dwellings and reduce human-vector contact [2,3]. These methods include, but are not limited to, the topical application of repellents, hand protection before touching some vectors, insecticide spraying, using window and door screens, and eliminating debris around the house. Despite the availability of preventive measures, there remains a gap in public health knowledge regarding why some individuals and households adopt these measures while others do not. To address this gap, it is essential to foster collaboration with local communities in which local knowledge and practices are appropriately integrated into research as well as control and prevention efforts.

One Health traditionally focuses on integrating human, environmental, and animal health. Building on this, several scholars have advocated for approaches to One Health that go beyond historical ideas tied to colonial policies, where people and some animals were seen as subjects to be managed for the sake of public health [4]. These approaches also value people’s knowledge, multispecies interactions, and how local communities manage their environment, recognizing each as crucial contributors to resolving health concerns [4]. Acknowledging that human-animal relationships are vital to health systems helps explain why people adopt or avoid practices that minimize contact with vectors.

We focus on two endemic, co-occurring vector-borne zoonotic diseases in Latin America: American Cutaneous Leishmaniasis (ACL) and Chagas disease (CD). ACL is transmitted to humans through the bite of infected sandflies that introduce *Leishmania* parasites into the skin, while CD is mainly transmitted through contact with the feces of triatomines (also known as kissing bugs) infected with *Trypanosoma cruzi* parasites [5,6]. In general, the scientific literature does not find a clear relationship between a person’s awareness or understanding of ACL and CD and their behavior that may reduce exposure to disease vectors [7]. Instead, research points to ecological, demographic (gender and age), socioeconomic, and cultural factors that influence people’s preventive practices for leishmaniases [8–10]. Similarly, the degree to which people practice behaviors to prevent CD has been linked to social or cultural factors like division of labor, where women spend more time cleaning patios or corrals than men, or differing disease perception, where people do not consider triatomine bugs or CD as a health threat [11,12].

Our recent review [7] highlights that social science research on leishmaniases and CD has primarily focused on people’s knowledge, attitudes, and practices (KAP surveys) and socioeconomic status (SES) in relation to pathogen transmission. While both lines of research shed light on the interaction between people and these pathogens, they also reveal certain limitations. For instance, KAP-oriented research may incorrectly assume that increased knowledge about a disease will automatically lead to changes in behavior [13]. Additionally, the tendency to oversimplify leishmaniases and CD as issues of “poverty” or low-income levels along with the assumption that low income correlates with lower knowledge and less disease prevention practices, may incorrectly imply that wealth and knowledge are correlated – without evidence to support that assumption [7]. Further attention to the specific pathways that connect behaviors that prevent disease with knowledge of a disease or SES will help strengthen not only strategies for the control of CD and ACL but also other neglected diseases.

Beyond knowledge or awareness of a disease and SES, individuals’ feelings towards vectors and perceptions about their health may help to explain risk-reducing practices. One attempt to evaluate health-related perceptions is the Multidimensional Health Locus of Control (MHLC) instrument, which assesses perceptions of control over one’s health [14]. The MHLC instrument divides people’s perceptions around who or what can control one’s health into three dimensions: internal (believing personal actions influence health), external powerful others (attributing health outcomes to authorities like doctors), and external chance (viewing health outcomes as random). Perceived control over health, or the idea of a ‘locus of control’, is one of the most studied and influential ideas in psychology [15]. A general pattern exists, indicating that individuals with higher internal control scores are more likely to engage in healthy behaviors, such as regular physical activity and a balanced diet [16]. However, the majority of research has been conducted in the US, Europe, and other high-income contexts [15,16] and applied to non-communicable diseases, which is limiting the understanding of the relationship between perceptions of health control and behaviors, especially for vector-borne diseases. For example, in Haiti, Kaiser 2024 [17] found an association between external control scores and mental health. In rural Bolivia, Alami et al. 2018 [18] also found that external control, especially powerful others, was associated with using pharmaceutical treatments, while there was no association between internal control and pharmaceutical treatments. Both of these points to the possibility that health and treatment decisions may be influenced by perceptions of the links between behaviors and their ability to make a difference. Understanding where individuals position themselves within these domains can clarify their motivations and barriers to adopting protective behaviors against vector-borne diseases. For example, those with a strong internal locus of control may take more personal responsibility, while those with a stronger external locus may respond more to guidance from health authorities or community leaders.

In this study, we assessed the association between household contextual elements, such as respondents’ sex, wealth, knowledge concerning ACL and CD, feelings about insects, and perceptions of control over their health (i.e., MHLC) and personal and property-based behaviors that may minimize contact with disease vectors—and, for one outcome, specifically to CD vectors. Our findings suggest links between wealth, people’s feelings toward insects, and their perception of control over health outcomes, with behaviors that may reduce or increase interactions with disease vectors. This research underscores the importance of addressing more comprehensive household contexts that encompass the complex entanglements of humans, animals, and their environment in promoting effective public health interventions to control and prevent vector-borne diseases.

## Methods

### Ethics statement

This research was carried out during 2022–2023 in Coclé Province, Republic of Panama. The project was reviewed and approved by the Bioethics Committee (Comité de Bioética de la Investigación) of the Instituto Conmemorativo Gorgas de Estudios de la Salud (#103/CBI/ICGES/22) and the University of Georgia IRB (#00000740). All participants were adults over 18 who read and signed informed consent describing the research aims before the survey.

### Settings

We conducted 204 household surveys in 12 rural communities within Coclé Province. The 12 research communities were selected to capture variation in the land use matrix of this region (pasture, mixed secondary forest, primary forest, etc.) of Panama, where ACL and CD are endemic. The interviews were conducted as part of a larger project on land use change and zoonotic disease risk that uses dogs as sentinels for CD and ACL.

Coclé is one of Panama’s central provinces, encompassing both urban, rural, and protected areas. The provincial capital, Penonomé, is approximately 150 km from Panama City. Coclé is one of the Panamanian provinces with the highest reported number of ACL cases [19]. Although there is no available data on the seroprevalence of CD in the communities we surveyed, a small number of CD cases have been documented in people in the Province of Coclé. In addition, interviews performed by our team with community health leaders in Coclé indicated that the COVID-19 pandemic had disrupted vector control efforts in the study area because of the focus on COVID-19.

According to the 2023 Instituto Nacional de Estadística y Censo of Panamá [20] Coclé has a population exceeding 268,000 inhabitants. The predominant economic activities in the province include agriculture, livestock farming, hunting, forestry, fishing, and related services [21]. Sociodemographic data reveal a median monthly income of employed individuals aged 10 and older of USD 400 [22] and a relatively low rate of non-readers (2.5%) among people over the age of 10 years [23]. Among the population of Coclé aged over four years (253,495 individuals), 3.4% had no degree, 36.6% had completed primary school, 40% had completed high school, and 14% had completed some degree of college education [24].

### Sampling strategy and data collection

We used a systematic sampling with implicit stratification to select households in each study community and account for spatial dispersion among the households throughout the geographical area of the communities, i.e., to sample communities in the center and periphery of the community borders [25–27]. The geographical location of each household was identified across 12 communities using georeferenced maps derived from the Ninth Panama Census of Population and Households 2010 [28]. We sorted households from north to south to establish a spatial order and divided the target number of households to be sampled (n = 24 target household sample size based on a parallel epidemiologic study of vector-borne pathogens in household dogs assuming a 2.5 average dog/household number) by the total number of households in the community to get the sampling interval (k). We chose the first (most ‘northern’) house to be sampled by selecting a random number between 1 and the sampling interval (k) and then chose every kth house after that first house. When we visited the communities abandoned or unoccupied houses in our original list were replaced with the nearest inhabited ones.

Primary interview data were collected by at least two teams—each composed of two interviewers—during household visits. The teams completed the survey in four visits to Coclé province: May and November 2022, and March and December 2023, three communities visited in each trip. At each selected household, the team explained the research and survey methods and obtained signed consent from an adult household member who agreed to participate in the interview. All interviews were conducted by a native speaker of Panamanian Spanish who noted responses on a paper version of the interview. A second interviewer simultaneously entered anonymized responses into a digital data collection tool (Kobo Toolbox). Households were assigned an alphanumeric code on all interview information to ensure confidentiality and anonymity. The interviewers compared responses across paper and digital versions at the end of the day and, when there was disagreement, the paper version was prioritized (see full survey in S1 Appendix).

We asked study participants about household demography, material wealth, household condition, their behaviors and health perception, feelings regarding insects, and understanding of ACL and CD. We based outcome variables on two personal and three property-based behaviors that may reduce exposure to disease vectors. The personal action questions were: (i) “Do you use repellents or lotion to protect yourself against insect bites?” and (ii) “Would you use any kind of protection before having contact with kissing bugs?”. Both questions had three response options (never, sometimes, or always), which were dichotomized into no/yes (0,1), with “no” indicating never and “yes” indicating sometimes or always. The property-based action questions were: (i) “Do you have screens on your windows/doors?”, (ii) “Have you used any treatment to prevent insects on the walls and floors of your home in the last 6 months?”, and (iii) “Do you have debris accumulated around your house?” The first question had four response options (screens on both windows and doors, only windows, only doors, some windows and/or doors), which were dichotomized into no/yes (0,1), with “no screens” indicating the absence of screens and the remaining answers indicating “screens on some/all windows and/or doors” indicating their use. The second and third questions had two response options (no/yes), coded as 0,1.

We included the following predictor variables in the analysis:

i) Household demography – specifically, the respondent’s sex, coded as 0 = female and 1 = male.
ii) Knowledge of ACL and CD – assessed through a series of questions: “Have you heard about leishmaniasis or any term listed?”, “Have you heard about Chagas disease?”, and “Do you recognize any of these kissing bugs (after some vectors that occur in Panama were shown)?”, each was coded as 0 = no and 1 = yes. Residents who had heard about ACL were also asked “Has anyone in your home had ACL?” and each answer was coded as no/yes (0,1). To evaluate understanding of disease transmission, participants were asked, “Do you know how leishmaniasis is transmitted?” Responses were coded as understood ACL is transmitted by an insect [1] if they answered “by an insect.” All other responses—such as “by a worm,” “by lack of hygiene,” or “do not know”—were coded as 0, indicating they did not know.
iii) Feelings regarding insects – assessed through two questions: “Do you feel bothered by insects indoors?” and “Do you feel bothered by insects outdoors?” Both were coded as 0 = no and 1 = yes.
iv) Perceived control over health outcomes – measured using the Multidimensional Health Locus of Control (MHLC) scale, categorized into three dimensions internal, external (powerful others), or external chance (see S1 Table for full details).
v) Wealth – assessed based on ownership of consumer goods and agricultural assets. To evaluate households’ wealth, respondents were asked to indicate ownership of common items and animals and the quantity of each owned item/animal. We developed a list of items that would capture variation in household wealth by modifying standard lists through discussions with team members who live or work extensively in the area. The final list of items and animals included bicycles, motorcycles, cars, radios, televisions, cable television, internet access, landline phones, cell phones, refrigerators, washing machines, sewing machines, computers, gas stoves, fans, gas generators, grinders, wooden bed frames, saddles, chickens, pigs, ducks, horses, and cattle. Additionally, respondents were queried about the presence of a toilet, latrine, and electricity meter for the household, with responses recorded as binary (yes/no) options (see full survey in S1 Appendix).

### Data analysis

We used Factor Analysis of Mixed Data (FAMD) via the Factoshiny package [29] in RStudio (Version 2024.04.2 + 764) to analyze the mixed data and create measures of wealth. FAMD is a multivariate statistical method designed to accommodate quantitative and qualitative data. It integrates principal component analysis (PCA) for quantitative variables with multiple correspondence analysis (MCA) for qualitative variables. This method facilitates the identification of underlying factors, whether quantitative or qualitative, that account for the variability observed in the dataset.

The FAMD analysis identified two principal dimensions that collectively accounted for 33% of the total variance in the dataset. This value is greater than the reference value that equals 18.28%, the variability explained by this plane is thus significant (the reference value is the 0.95-quantile of the variance percentages distribution obtained by simulating 3991 data tables of equivalent size on the basis of a normal distribution). The first dimension, what we have labeled “Consumer Goods and Appliances”, captures the variability in ownership of common household consumer goods and electrical appliances. Variables such as cellphones, cars, televisions, refrigerators, washing machines, internet access, and the presence of an electricity meter exhibited higher loadings on this dimension. Therefore, a higher score on this dimension indicates that households possess greater numbers of consumer goods and appliances. The second dimension, which we refer to as Agricultural Wealth, is characterized by assets associated with horticultural or agriculture-centered rural lifestyles. Variables such as saddles, horses, and cattle showed higher loadings on this dimension. In this region of Panama, horses are primarily associated with cattle ranching instead of other reasons for owning horses (leisure, breeding, sport). Therefore, we interpreted higher scores on the second dimension to be associated with an independent source of wealth- animal and farming wealth. A factor loading score for each of the two dimensions was calculated for each household in FactoShiny. We then used these factor-loading scores as our measures of wealth in the analysis.

We used the Multidimensional Health Locus of Control (MHLC) instrument to capture residents’ perception of control over their health outcomes. The MHLC is a commonly used survey instrument designed to assess individuals’ perceptions of their health control, attributing it to three distinct dimensions of control: internal (themselves), external powerful others (such as doctors and family), and external chance [14]. Each dimension is represented by six statements regarding respondents’ health conditions, which assess their perceptions of health. Respondents indicate their level of agreement with each statement on a scale from 1 (strongly disagree) to 5 (strongly agree). Higher scores reflect greater agreement, while lower scores indicate greater disagreement (see S1 Table). Entries in the database include a score for each statement ranging from 1 to 5. For each dimension, a composite variable was calculated by summing the scores for the six statements within that dimension, with the resulting scores ranging from 6 (disagreement with the idea that the domain influenced health) to 30 (strong agreement that the domain influenced health).

We used logistic regression models to analyze the effects of respondents’ sex, wealth (consumer goods/appliances and agricultural wealth), knowledge about ACL and CD, feelings about insects, and perception of their health outcomes (MHLC) on personal (use of repellents, and hand protection before touching a CD vector) and property-based behaviors (use of insecticides in the house, use of windows/doors screens, and cleaning peridomestic debris) that may reduce exposure to disease vectors. All models were performed on RStudio (Version 2024.04.2 + 764).

## Results

### General results

A total of 204 adults participated in our survey. Most participants were women (59%), older than 54 years of age (Median age 54 years; IQR 42–63), and had completed primary or middle school (60%). Most householders reported being bothered by insects indoors (69%) and outdoors (77%). Awareness of ACL was high (87% had heard about the disease or other term/s associated with ACL), but only 66% reported knowing ACL is transmitted by insects. On the other hand, only 11% of householders were aware of CD, although 72% confirmed they had seen at least one CD vector (*Rhodnius pallescens*, *Triatoma dimidiata*, and/or *Panstrongylus rufotuberculatus*) when specimens were displayed to respondents. The median responses for each domain in the MHLC instrument indicated agreement with ideas that powerful others (median 23, IQR 21–24), self-control (median 23, IQR 21–24), and chance (median 21, IQR 18–23) influenced respondents’ health outcomes.

People reported that they regularly used measures to protect both themselves (personal) and their households (property protection) from insects. Slightly more than half of the households reported that they have (always or sometimes) used repellents (51%). Most respondents (71%) reported using some kind of protection before touching a CD vector (Table 1). Fifty-nine percent of the households surveyed reported using window/door screens in some or all of their windows and/or doors. Nearly half of the participants confirmed that they always or sometimes use insecticides or other products to kill insects on their walls and/or floors. Sixty-three percent of respondents reported the presence of debris around their residences (Table 2).

**Table 1:** Description of the studied population (demographics, wealth, knowledge about American Cutaneous Leishmaniasis [ACL] and Chagas disease [CD], nuisance feelings toward vectors indoors and outdoors, and perceptions of control over their health outcomes) by personal protective behaviors (use of repellents and use of protection before touching a kissing bug).

Predictors	Do you use repellents or lotion to protect you against insects’ bite?				Would you use any kind of protection before having contact with kissing bugs?			
	Always/ Sometimes, N = 104^1^	Never, N = 100^1^	Total^1^	p-value^2^	Always/ Sometimes, N = 104^1^	Never/ Not sure, N = 43^1^	Total^1^	p-value^2^
**Age**	52 (22)	55 (22)	54 (21)	0.13	54 (16)	54 (18)	54 (16)	0.95
**Sex**				0.51				0.63
Female	64 (62%)	57 (57%)	121 (59%)		56 (54%)	25 (58%)	81 (55%)	
Male	40 (38%)	43 (43%)	83 (41%)		48 (46%)	18 (42%)	66 (45%)	
**Education**				0.19				0.04
No education/ Incomplete primary school	29 (28%)	24 (24%)	53 (26%)		25 (24%)	19 (44%)	44 (30%)	
Primary only/Middle school	57 (55%)	66 (66%)	123 (60%)		67 (64%)	19 (44%)	86 (59%)	
High school/University	18 (17%)	10 (10%)	28 (14%)		12 (12%)	5 (12%)	17 (12%)	
**Consumer goods and appliances**	0.22 (3.61)	-0.40 (2.99)	-0.01 (3.44)	0.13	-0.04 (2.27)	-0.61 (2.27)	-0.21 (2.28)	0.17
**Agricultural wealth**	-0.35 (1.29)	-0.26 (1.26)	-0.31 (1.29)	0.94	-0.16 (1.05)	0.16 (1.17)	-0.06 (1.09)	0.13
**Had heard about ACL or any other term**				0.17				0.51
No	10 (9.6%)	16 (16%)	26 (13%)		10 (9.6%)	2 (4.7%)	12 (8.2%)	
Yes	94 (90%)	84 (84%)	178 (87%)		94 (90%)	41 (95%)	135 (92%)	
**Understood ACL is transmitted by an insect** ^a^				0.48				0.27
No	30 (32%)	31 (37%)	61 (34%)		32 (34%)	10 (24%)	42 (31%)	
Yes	64 (68%)	53 (63%)	117 (66%)		62 (66%)	31 (76%)	93 (69%)	
No answer ^b^	10	16	26		10	2	12	
**Had a case of ACL in the household**				0.50				0.03
No/Does not know	54 (57%)	44 (52%)	98 (55%)		56 (60%)	16 (39%)	72 (53%)	
Yes	40 (43%)	40 (48%)	80 (45%)		38 (40%)	25 (61%)	63 (47%)	
No answer ^b^	10	16	26		10	2	12	
**Had heard about CD**				0.58				0.76
No	94 (90%)	88 (88%)	182 (89%)		90 (87%)	38 (88%)	128 (87%)	
Yes	10 (9.6%)	12 (12%)	22 (11%)		14 (13%)	5 (12%)	19 (13%)	
**Recognize kissing bugs**				0.12				--
No	34 (33%)	23 (23%)	57 (28%)		--	--	--	
Yes	70 (67%)	77 (77%)	147 (72%)		104 (100%)	43 (98%)	147 (100%)	
**Bothered by insects indoors**				<0.001				0.91
No	20 (19%)	44 (44%)	64 (31%)		30 (29%)	12 (28%)	42 (29%)	
Yes	84 (81%)	56 (56%)	140 (69%)		74 (71%)	31 (72%)	105 (71%)	
**Bothered by insects outdoors**				0.00				0.78
No	14 (13%)	32 (32%)	46 (23%)		22 (21%)	10 (23%)	32 (22%)	
Yes	90 (87%)	68 (68%)	158 (77%)		82 (79%)	33 (77%)	115 (78%)	
**Powerful others**	22.0 (3.3)	23.0 (4.0)	23.0 (4.0)	0.02	22.7 (2.8)	23.7 (3.6)	23.0 (3.1)	0.15
**Internal**	23.00 (3.00)	23.00 (3.00)	23.00 (3.0)	0.84	22.5 (2.6)	24.2 (3.3)	23.0 (2.9)	0.00
**Chance**	20.5 (4.3)	21.0 (4.3)	21.0 (5.0)	0.08	20.2 (3.9)	21.2 (4.5)	20.5 (4.1)	0.44

^1^Median (IQR), n (%);

^2^Wilcoxon rank sum test, Pearson’s Chi-squared test, Fisher’s exact test.

-- is displayed because the question about using protection before touching a kissing bug is asked only to respondents who previously answered that they recognize a kissing bug.

^a^Respondents were asked “Do you know how leishmaniasis is transmitted?” and were provided with a list of options to choose from. Those who answered “It is transmitted by the bite of an insect” were considered to understand the correct mode of transmission and were coded as “yes” (1), while all other incorrect options selected by the respondents were coded as “no” (0), indicating a lack of understanding.

^b^No answer refers to those who had responded “no” to the question, “Have you heard about leishmaniasis or any other term listed?” In these cases, respondents were not asked about the mode of transmission of leishmaniasis or the occurrence of previous cases in the household because they hadn’t heard about the disease. For the logistic models, they were coded as no (0).

**Table 2:** Description of the studied population (demographics, wealth, knowledge about American Cutaneous Leishmaniasis [ACL] and Chagas disease [CD], nuisance feelings toward vectors indoors and outdoors, and perceptions of control over their health outcomes) by property-based protective behaviors (use of window/door screens, use of insecticide on walls and floors, and presence of debris around the house).

Predictors	Do you have screens on your windows/doors?			Have you used any treatment to prevent insects on the walls and floors of your home in the last 6 months?			Do you have debris accumulated around your house?			Total^1^
	No screens, N = 120^1^	Some windows and doors, N = 84^1^	p-value^2^	No, N = 97^1^	Yes, N = 107^1^	p-value^2^	No, N = 128^1^	Yes, N = 76^1^	p-value^2^	
**Age**	52 (16)	54 (17)	0.13	52 (17)	54 (16)	0.42	54 (17)	51 (16)	0.48	53 (16)
**Sex**			0.73			0.89			0.23	
Female	70 (58%)	51 (61%)		58 (60%)	63 (59%)		80 (63%)	41 (54%)		121 (59%)
Male	50 (42%)	33 (39%)		39 (40%)	44 (41%)		48 (38%)	35 (46%)		83 (41%)
**Education**			0.01			0.55			0.24	
High school/University	10 (8.3%)	18 (21%)		13 (13%)	15 (14%)		14 (11%)	14 (18%)		28 (14%)
No education/ Incomplete primary	37 (31%)	16 (19%)		22 (23%)	31 (29%)		32 (25%)	21 (28%)		53 (26%)
Primary only/ Middle school	73 (61%)	50 (60%)		62 (64%)	61 (57%)		82 (64%)	41 (54%)		123 (60%)
**Consumer goods and appliances**	-0.66 (1.91)	0.95 (2.25)	<0.001	0.10 (2.18)	-0.09 (2.23)	0.55	0.05 (2.15)	-0.09 (2.29)	0.64	0.00 (2.20)
**Agricultural wealth**	0.03 (1.54)	-0.05 (1.21)	0.95	0.03 (1.68)	-0.03 (1.11)	0.69	0.02 (1.52)	-0.04 (1.21)	0.98	0.00 (1.41)
**Had heard about ACL or any other term**			0.58			0.35			0.46	
No	14 (12%)	12 (14%)		18 (19%)	8 (7.5%)		18 (14%)	8 (11%)		26 (13%)
Yes	106 (88%)	72 (86%)		79 (81%)	99 (93%)		110 (86%)	68 (89%)		178 (87%)
**Understood ACL is transmitted by an insect** ^a^			0.24			0.35			0.92	
No	40 (38%)	21 (29%)		30 (38%)	31 (31%)		38 (35%)	23 (34%)		61 (34%)
Yes	66 (62%)	51 (71%)		49 (62%)	68 (69%)		72 (65%)	45 (66%)		117 (66%)
No answer ^b^	14	12		18	8		18	8		26
**Had a case of ACL in the household**			0.47			0.65			0.09	
No/Does not know	56 (53%)	42 (58%)		42 (53%)	56 (57%)		66 (60%)	32 (47%)		98 (55%)
Yes	50 (47%)	30 (42%)		37 (47%)	43 (43%)		44 (40%)	36 (53%)		80 (45%)
No answer ^b^	14	12		18	8		18	8		26
**Had heard about CD**			0.18			0.84			0.40	
No	110 (92%)	72 (86%)		87 (90%)	95 (89%)		116 (91%)	66 (87%)		182 (89%)
Yes	10 (8.3%)	12 (14%)		10 (10%)	12 (11%)		12 (9.4%)	10 (13%)		22 (11%)
**Recognize kissing bugs**			0.87			0.97			0.37	
No	33 (28%)	24 (29%)		27 (28%)	30 (28%)		33 (26%)	24 (32%)		57 (28%)
Yes	87 (73%)	60 (71%)		70 (72%)	77 (72%)		95 (74%)	52 (68%)		147 (72%)
**Bothered by insects indoors**			0.68			0.90			0.01	
No	39 (33%)	25 (30%)		30 (31%)	34 (32%)		32 (25%)	32 (42%)		64 (31%)
Yes	81 (68%)	59 (70%)		67 (69%)	73 (68%)		96 (75%)	44 (58%)		140 (69%)
**Bothered by insects outdoors**			0.18			0.97			0.18	
No	31 (26%)	15 (18%)		22 (23%)	24 (22%)		25 (20%)	21 (28%)		46 (23%)
Yes	89 (74%)	69 (82%)		75 (77%)	83 (78%)		103 (80%)	55 (72%)		158 (77%)
**Powerful others**	23.3 (3.3)	22.6 (3.4)	0.28	23.1 (3.3)	22.9 (3.4)	0.66	22.9 (3.2)	23.1 (3.7)	0.96	23.0 (3.3)
**Internal**	22.88 (2.87)	23.17 (2.93)	0.22	22.95 (2.84)	23.04 (2.95)	0.87	22.99 (2.94)	23.00 (2.82)	0.76	23.00 (2.89)
**Chance**	20.3 (4.0)	20.3 (4.0)	0.88	20.7 (3.5)	19.9 (4.4)	0.21	20.7 (3.7)	19.6 (4.4)	0.04	20.3 (4.0)

^1^Median (IQR), n (%);

^2^Wilcoxon rank sum test, Pearson’s Chi-squared test.

^a^Respondents were asked “Do you know how leishmaniasis is transmitted?” and were provided with a list of options to choose from. Those who answered, “It is transmitted by the bite of an insect” were considered to understand the correct mode of transmission and were coded as “yes” (1), while all other incorrect options selected by the respondents were coded as “no” (0), indicating a lack of understanding.

^b^No answer refers to those who had responded “no” to the question “Have you heard about leishmaniasis, or any other term listed?” In these cases, respondents were not asked about the mode of transmission of leishmaniasis or the occurrence of previous cases in the household because they hadn’t heard about the disease. For the logistic models, they were coded as ‘no’ (0).

### Personal protective behaviors

**Fig 1:**
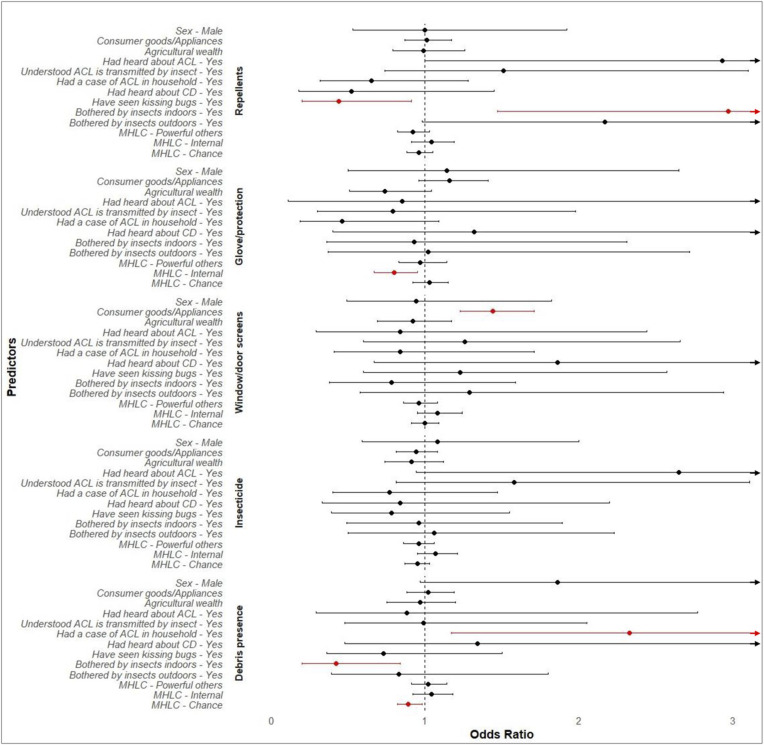
Association between elements of the household context and protective behaviors as estimated by logistic regression models. The figure displays odds ratios (OR) and 95% confidence intervals (CI) from logistic regression models. Predictors include demographics (sex), wealth (consumer goods/appliances and agricultural wealth), knowledge about American Cutaneous Leishmaniasis (ACL) and Chagas disease (CD), feelings of nuisance toward insects (indoors and outdoors), and perceptions of control over health (powerful others, internal, and chance), as measured by the Multidimensional Health Locus of Control (MHLC). Protective behaviors are categorized into personal (e.g., use of insect repellent and use of hand protection before handling a kissing bug) and property-based (e.g., use of insecticides, use of window/door screens, and presence of peridomestic debris). For binary predictors, the OR reflects the odds of the protective behavior among individuals in the category shown in the figure (‘Yes’ or ‘Male’) relative to the reference group (‘No’ or ‘Female’). OR greater than 1 indicates increased odds of engaging in protective behavior, while values less than 1 indicate decreased odds. Red indicates the 95% CI does not include 1. Arrows indicate 95% CI extends beyond the plotted range.

There are mixed associations between persons’ protective behaviors and their household context*.* For example, individuals who reported feeling bothered by insects inside their homes were nearly three times more likely to use repellent compared to those who reported that insects did not bother them (odds ratio [OR]: 2.97; 95% confidence interval [CI]: 1.47-6.20). A similar result was observed by those who reported feeling bothered by insects outdoors, although CI 95% included value 1 (OR: 2.17; CI 95% [CI]: 0.98-4.92). In contrast, individuals who reported that they had seen a kissing bug were approximately 56% less likely to use repellent than those who had not seen one (OR: 0.44; 95% CI: 0.20-0.91). For each one-unit increase in the belief that one has control over their health outcomes, individuals were approximately 20% less likely to use protection before touching a kissing bug (OR: 0.80; 95% CI: 0.67-0.95) (S2 Table and Fig 1). There were no significant relationships between the respondents’ sex and the assessed protective behaviors or measures of household wealth and their reported use of personal protective measures against insects.

### Property-based protective behaviors

We found that the association between household measures of wealth and reported property-protective behaviors was related to the presence of screens in windows and doors. Households that ranked higher on the “Consumer Goods and Appliances” factor were nearly 1.5 times more likely to have window or door screens (OR: 1.44; 95% CI: 1.23-1.7). There were no other associations between wealth and property-based protective behaviors.

Our results show possible links between peridomestic debris and the household context. Households that reported a history of ACL cases were 2.33 times more likely to have debris accumulated around the house than those without such a history (OR: 2.33; 95% CI: 1.17-4.75). In contrast, having debris around a household was less likely (approximately 58%) in households that reported feeling bothered by insects indoors (OR: 0.42; 95% CI: 0.20-0.84) and in households where the respondents agreed more strongly that their health outcomes were due to chance (OR: 0.89; 95% CI: 0.82-0.98). Here, a one-unit increase in agreement with the idea that health outcomes were shaped by chance, households were 11% less likely to have debris around the house. No significant associations were found between the use of insecticides or other treatments for insects on walls and floors and the predictors examined (S3 Table and Fig 1).

## Discussion

The results of this study shed light on the various factors that influence the adoption of personal and property-based protective measures against insect vectors in the Coclé Province, Panama. Our findings align with existing literature that identifies wealth measures and some knowledge about ACL and CD as important drivers of vector-borne disease prevention practices [7] and adds nuanced insights into the interaction between household contextual elements and preventive behaviors. First, in this area where ACL and CD are endemic, people reported knowledge of diseases and that they regularly, but not always, used measures to protect both themselves (personal) and their households (property protection) from insects. Second, the way that people feel about insects seems to be related to both personal and property-level insect preventive behavior. Third, ideas about control over health were related to some protective measures in unexpected and counterintuitive ways. Finally, the lack of clear association between measures of wealth and prevention behaviors, except for the use of house screens, suggests that wealth may not always be central to why people choose to use personal or property-level protection tools against vector-borne diseases. While the specific details of the study cannot be generalized beyond the study area, our finding that it may be worthwhile to consider more complex associations between household contextual factors, control over health, and health-related behaviors can be applied more widely.

A body of research assesses how people understand diseases in areas where vector-borne pathogens are endemic. Our finding that, in this endemic area, 87% of participants were aware of ACL, but only 11% of participants were familiar with CD is consistent with other research on both diseases [7]. The symptoms of ACL are highly visible, non-healing skin ulcers, which have led to important local knowledge about the nature of this disease [7,30,31]. In contrast, the kissing bugs, vectors responsible for spreading CD, are “charismatic” and therefore well-known in our research, but their association with the disease is even more limited than in other parts of Panama where health education programs have been carried out [32] – probably because many people were unaware of the relationship between contact with kissing bugs and the underreported and non-visible cases of CD. Educational campaigns and public health programs aimed at raising awareness of CD and its vectors could improve public understanding by linking visible vectors to the disease. Such programs could improve both awareness and early detection efforts and, potentially, preventive behaviors, in areas where these diseases are endemic.

To our knowledge, this is one of a handful of studies that explores people’s feelings about insects in and around their household and their use of insect-protection measures [7]. Our results may help to explain some variation in why people invest time, money, or risk exposure to chemicals in order to avoid insects. For instance, the association between consumer goods/appliances and property-based protective actions, such as the use of window/door screens, highlights the relevance of material wealth to implement some effective household interventions. Simultaneously, this response highlights people do use repellent or insecticide when they choose to, offering a counterpoint to the deficit-focused narrative that attributes non-use solely to a lack of household financial resources [9,33]. Indeed, our results suggest that people who reported being bothered by insects in their households were more likely to use repellent and maintain cleaner surroundings with less debris, reinforcing the lack of association between wealth and other personal and property-based actions of vector avoidance in this study. While our study design does not allow us to distinguish between the presence of insects and reports of being bothered by insects (e.g., if people aren’t bothered does it mean that there are no insects or they aren’t bothered by the insects present), it does suggest that it may be an oversimplification to assume that insects are everywhere and continually bothering people in rural regions.

The focus on limiting insect-human interactions is dominant in vector control, and current efforts to control vector-borne diseases have also been challenged by several scholars [4,34]. Furthermore, scholars [35–38] have pointed to a gradual transition in how global health conceptualizes the relationship between people, insects, and animals. They argue that with the rise of the Emerging Infectious Diseases framework in the 21st century, there was also a move away from viewing animals and insects as carriers of disease that would result in an occasional infection with limited risks toward a situation where emergence is inevitable and ongoing [35–38]. Although insect vector control remains central to disease control, our results caution that it is possible that people’s desire and willingness to limit interactions with insects may extend beyond the desire to control infectious diseases. In fact, ethnobiological research on how people view insects shows diverse perceptions, including recognizing the many points of interaction with humans ranging from sources of pain and injury, sources of contagion, possible food sources, agricultural friends or foes, and organisms with important social, religious, and cultural significance [39–44].

This study also underscores the importance of perception and feelings in shaping disease prevention behaviors. For instance, individuals who were bothered by insects were more likely to use repellents, yet those who had seen kissing bugs were less likely to use repellents. Similarly, participants who believe they have control over their health were less likely to use protection before touching a kissing bug, whereas those who reported that their health issues happen by chance reported less debris around their house. These counterintuitive results contrast with the general findings that internal locus of control is positively associated with practicing preventive behaviors [16]. This may be partly explained by the idea that perceptions about control over health can also be shaped by how people prioritize their health over other concerns, such as economic security, family responsibilities, or social obligations [45]. Here, a hierarchies of risk model [46] may help to understand how people respond to health interventions in contexts where multiple diseases and other concerns (economic, family concerns, stigma, etc.) exist. While we did not collect systematic survey data about risk perceptions, our observations are that health concerns are balanced with family and economic concerns in the study area. Our counterintuitive findings may, therefore, reflect an underestimation of disease risk by the households, possibly due to the prioritization of other concerns and diseases or a lack of recognition that kissing bugs can transmit disease-causing pathogens and that debris may increase the risk of vector-borne diseases. They may also reflect a relatively lower concern due to the chronic nature of the disease or possible misunderstanding or misinformation that occurs in areas with limited access to treatment. Another possible explanation is the overconfidence in internal control; those with a high internal MHLC might believe they are already in control of their health and underestimate the need for further preventive behaviors. Additionally, in some societies or contexts, preventive behaviors, such as keeping the area around the house free of debris, may be more strongly influenced by cultural or situational factors, such as having fewer women in the household, who are often responsible for domestic cleaning. Furthermore, some people may find it difficult to keep their surroundings tidy due to being too busy, feeling depressed, or being overwhelmed by other challenges (e.g., family emergencies, un/underemployment). Finally, the lack of research in rural, health resource-limited areas points to the need for further research unpacking the associations between behavior and ideas of control over health outcomes.

This study, while providing valuable insights into the factors that influence vector-borne disease prevention behaviors, is subject to several limitations. First, the study relied on self-reported data, which may introduce recall bias. However, we minimized this risk by using well-established, validated questionnaires specifically designed to reduce recall issues. Additionally, trained interviewers framed the questions in neutral terms to prevent leading participants toward socially desirable responses and to avoid emotionally charged or judgmental language. Second, we rely on people reporting they were bothered by insects without direct environmental measures to confirm insect abundance or distinguish between species. This means we are unable to distinguish if feelings of being bothered by insects are related to the actual exposure to insects or subjective experiences of being “bothered” by insects. However, in our survey, participants’ discomfort with insects indoors appears to be primarily caused by mosquitoes (88%; 123/140), followed by sandflies (29%; 29/140). With our focus on the association between being bothered and behavior, the actual presence of specific vectors is less important. Any insect-related discomfort may still promote protective behaviors, which could, in turn, indirectly reduce exposure to ACL and CD vectors and other vector-borne diseases in the region. Third, while this research provides a broad understanding of protective behaviors, it does not delve into all potential factors that could influence behaviors, such as community norms or economic factors, that may influence decision-making around personal and household-disease prevention practices. For instance, prior experience with a disease may influence perceived utility of preventive actions—individuals who have already been infected may view such measures as less necessary. However, none of the respondents reported having either CD or ACL at the time of the interview. Fourth, the findings related to MHLC necessitate further investigation to explore the underlying reasons behind individuals’ ideas of health control and risk related to vector-borne diseases. Finally, as a cross-sectional study, our results cannot be interpreted as causal associations.

Our study recognizes the complex relationships between humans, animals, and ecosystems and demonstrates the value of a more nuanced understanding of multispecies interactions. Rather than viewing insects and other animals only as threats to human health, our results suggest there is value in including information about how people consider insects in their daily lives and the diverse roles animals and insects play in ecosystems. This is consistent with what proponents call an extended model of One Health [47] that challenges the dominant notion that all insects must be controlled, a concept rooted in colonial frameworks that associate insects (and other animals) with disease, uncleanliness, and backwardness [4,34]. By demonstrating that people’s reported use of insect control measures is related to their feelings of being bothered by insects, awareness of a disease, health perception, and household wealth demonstrates the value of paying careful attention to household and community values and understandings to avoid oversimplifying human-insect-animal interactions through efforts to control zoonotic diseases.

## Conclusion

In conclusion, the observed associations found in this study provide valuable insights into the complex factors associated with vector-borne disease prevention behavior in the Coclé Province, Panama, and, perhaps, in other areas of Latin America where CD and leishmaniasis are endemic. Our findings reinforce existing literature on the links between wealth and cost-related preventive behaviors (i.e., use of windows/doors screen), yet they also reveal nuanced interactions between the household context and behaviors to prevent vector-borne diseases. Notably, while many participants reported awareness of ACL and kissing bugs, knowledge of CD was less common, possibly reflecting the distinct nature and symptoms of these diseases.

This study highlights that, despite the general awareness and use of preventive measures such as insect repellent, there is variability in how these practices are employed. The association between nuisance from insects and the use of preventive measures suggests that individual experiences and feelings towards insects may be associated with some protective behaviors. Moreover, the lack of a clear link between measures of wealth and the use of other cost-related preventive behaviors (i.e., insecticides and repellents) suggests that factors beyond wealth, including cultural and situational contexts, play a role in preventive practices.

Interestingly, our findings also challenge some conventional assumptions about vector control. The recognition of CD vectors and the perception of control over health were associated with preventive behavior in unexpected ways, potentially reflecting a complex interplay between risk perception and preventive actions. For instance, those who felt more control over their health were less likely to engage in some preventive behaviors like the use of protection before having contact with a kissing bug, which may be influenced by an underestimation of disease risk or overconfidence in personal health control.

Overall, this study underscores the importance of considering the household context, beyond the knowledge of a specific disease or measures of wealth, in vector-borne disease prevention strategies in Coclé Province, Panama, and possibly in other rural areas where vector-borne diseases occur. While our findings cannot be interpreted as causal associations, they do point to possible future areas of research that go beyond standard assumptions that wealth, education, or knowledge of a disease will drive protective measures. This is consistent with calls for tailored public health interventions that also consider perceptions, feelings, value systems, and sociocultural contexts in shaping preventive behaviors. Further research should explore these relationships to refine and improve prevention efforts in areas where vector-borne diseases are endemic. Addressing these factors and understanding their interplay can enhance public health interventions and tailor them to the specific needs of affected communities.

## Supporting information

S1 Appendix Full Survey (Spanish and English Translation). This appendix contains the complete version of the questionnaire used in the study. The original questionnaire was administered in Spanish. An English translation is provided for reference, following the original structure and wording as closely as possible.
(DOCX)

S1 Table Multidimensional Health Locus of Control. Statements of internal, external powerful others, and external chance domains and scores ranging from strongly disagree to strongly agree.
(DOCX)

S2 Table Logistic regression models showing the association between elements of the household context—including demographics (sex), wealth (consumer goods/appliances and agricultural wealth), knowledge about American Cutaneous Leishmaniasis (ACL) and Chagas disease (CD), feelings of nuisance toward insects both indoors and outdoors, and perceptions of control over health (powerful others, internal, and chance), as measured by the Multidimensional Health Locus of Control (MHLC)—and personal protective behaviors (use of repellents and hand protection before touching a kissing bug). Odds ratios (OR), 95% confidence intervals (CI), and p-values are shown for each predictor variable across the two outcomes. For binary predictors, the odds ratio represents the odds of the outcome for the ‘Yes’ or ‘Male’ category, compared to the ‘No’ or ‘Female’ (reference) category.
(DOCX)

S3 Table Logistic regression models showing the association between elements of the household context—including demographics (sex), wealth (consumer goods/appliances and agricultural wealth), knowledge about American Cutaneous Leishmaniasis (ACL) and Chagas disease (CD), feelings of nuisance toward insects both indoors and outdoors, and perceptions of control over health (powerful others, internal, and chance), as measured by the Multidimensional Health Locus of Control (MHLC)—and property-based protective behaviors (use of window/door screens, use of insecticides, and presence of debris around the house). Odds ratios (OR), 95% confidence intervals (CI), and p-values are shown for each predictor variable across the three outcomes. For binary predictors, the odds ratio represents the odds of the outcome for the ‘Yes’ or ‘Male’ category, compared to the ‘No’ or ‘Female’ (reference) category.
(DOCX)

## References

[1] World Health Organization. Vector-borne diseases. 2024 [cited 2024 Oct 31]. https://www.who.int/news-room/fact-sheets/detail/vector-borne-diseases

[2] Cucunubá ZM, Gutiérrez-Romero SA, Ramírez JD, Velásquez-Ortiz N, Ceccarelli S, Parra-Henao G. The epidemiology of Chagas disease in the Americas. Lancet Reg Health – Am. 2024;37:100881. doi: 10.1016/j.lana.2024.100881 39474465 PMC11519694

[3] de Vries HJC, Schallig HD. Cutaneous Leishmaniasis: A 2022 Updated Narrative Review into Diagnosis and Management Developments. Am J Clin Dermatol. 2022;23(6):823–40. doi: 10.1007/s40257-022-00726-8 36103050 PMC9472198

[4] Lainé N, Morand S. Linking humans, their animals, and the environment again: a decolonized and more-than-human approach to “One Health.”. Parasite. 2020;27:55. doi: 10.1051/parasite/2020055 33141658 PMC7608982

[5] Ross R. Note on the bodies recently described by Leishman and Donovan. Br Med J. 1903;2(2237):1261–2. doi: 10.1136/bmj.2.2237.1261 20761169 PMC2514667

[6] Chagas C. Nova tripanozomiaze humana: estudos sobre a morfolojia e o ciclo evolutivo do Schizotrypanum cruzi n. gen., n. sp., ajente etiolojico de nova entidade morbida do homem. Mem Inst Oswaldo Cruz. 1909;1(2):159–218. doi: 10.1590/s0074-02761909000200008

[7] Brito RN, Tanner S, Runk JV, Hoyos J. Looking through the lens of social science approaches: A scoping review of leishmaniases and Chagas disease research. Acta Trop. 2024;249:107059. doi: 10.1016/j.actatropica.2023.107059 37918504

[8] J S D, Gupta AK, Veeri RB, Garapati P, Kumar R, Dhingra S, et al. Knowledge, attitude and practices towards visceral leishmaniasis among HIV patients: A cross-sectional study from Bihar, India. PLoS One. 2021;16(8):e0256239. doi: 10.1371/journal.pone.0256239 34404087 PMC8370793

[9] Pardo RH, Carvajal A, Ferro C, Davies CR. Effect of knowledge and economic status on sandfly control activities by householders at risk of cutaneous leishmaniasis in the subandean region of Huila department, Colombia. Biomedica. 2006;26 Suppl 1:167–79. 17361852

[10] Berhe R, Spigt M, Schneider F, Paintain L, Adera C, Nigusie A, et al. Understanding the risk perception of visceral leishmaniasis exposure and the acceptability of sandfly protection measures among migrant workers in the lowlands of Northwest Ethiopia: a health belief model perspective. BMC Public Health. 2022;22(1):989. doi: 10.1186/s12889-022-13406-3 35578331 PMC9112482

[11] Salm A, Gertsch J. Cultural perception of triatomine bugs and Chagas disease in Bolivia: a cross-sectional field study. Parasit Vectors. 2019;12(1):291. doi: 10.1186/s13071-019-3546-0 31182163 PMC6558697

[12] Ventura-Garcia L, Roura M, Pell C, Posada E, Gascón J, Aldasoro E, et al. Socio-Cultural Aspects of Chagas Disease: A Systematic Review of Qualitative Research. PLoS Negl Trop Dis. 2013;7(9):e2410. doi: 10.1371/journal.pntd.0002410 24069473 PMC3772024

[13] Launiala A. How much can a KAP survey tell us about people’s knowledge, attitudes and practices? Some observations from medical anthropology research on malaria in pregnancy in Malawi. Anthropol Matters. 2009;11(1):1–13. doi: 10.22582/am.v11i1.31

[14] Wallston KA, Wallston BS, DeVellis R. Development of the Multidimensional Health Locus of Control (MHLC) Scales. Health Educ Monogr. 1978;6(2):160–70. doi: 10.1177/109019817800600107 689890

[15] Infurna FJ, Reich JW, Reich JW, Infurna FJ. Perceived control: 50 years of innovation and another 50 to go. Perceived control: Theory, research, and practice in the first 50 years. Oxford University Press. 2016.

[16] Dogonchi M, Mohammadzadeh F, Moshki M. Investigating the Relationship between Health Locus of Control and Health Behaviors: A Systematic Review. Open Public Health Journal. 2022.

[17] Kaiser BN. Locus of Control and Mental Health: Human Variation Complicates a Well-Established Research Finding. Am J Hum Biol. 2024;36(12):e24147. doi: 10.1002/ajhb.24147 39143843 PMC11645869

[18] Alami S, Stieglitz J, Kaplan H, Gurven M. Low perceived control over health is associated with lower treatment uptake in a high mortality population of Bolivian forager-farmers. Soc Sci Med. 2018;200:156–65. doi: 10.1016/j.socscimed.2018.01.017 29421462 PMC5893402

[19] Miranda A del C, González KA, Samudio F, Pineda VJ, Calzada JE, Capitan-Barrios Z. Molecular identification of parasites causing cutaneous leishmaniasis in Panama. Am J Trop Med Hyg. 2021;104(4):1326–34. doi: 10.4269/ajtmh.20-1336 33432903 PMC8045627

[20] Instituto Nacional de Estadística y Censo. Resultados finales básicos XII censo nacional de población y VIII de vivienda 2023. Cuadro 1. Viviendas particulares ocupadas y población en la República, por sexo, según provincia y comarca indígena: censos 2010 y 2023. 2023. https://www.inec.gob.pa/publicaciones/Default3.aspx?ID_PUBLICACION=1199&ID_CATEGORIA=19&ID_SUBCATEGORIA=71

[21] Instituto Nacional de Estadística y Censo. Volumen IV Población Económicamente Activa. Cuadro 3. Población de 10 y Más Años de Edad Económicamente Activa en la República, por Categoría en la Actividad Económica, según Provincia, Comarca Indígena, Sexo y Grupos de Edad: Censos 2023. 2023. https://www.inec.gob.pa/publicaciones/Default3.aspx?ID_PUBLICACION=1226&ID_CATEGORIA=19&ID_SUBCATEGORIA=71

[22] Instituto Nacional de Estadística y Censo. Volumen IV Población Económicamente Activa. Cuadro 11. Población de 10 y Más Años de Edad Ocupada en la República, por Ingreso Mensual Percibido, según Provincia, Comarca Indígena, Sexo y Grupos de Edad: Censos 2023. 2023. https://www.inec.gob.pa/publicaciones/Default3.aspx?ID_PUBLICACION=1226&ID_CATEGORIA=19&ID_SUBCATEGORIA=71

[23] Instituto Nacional de Estadística y Censo. Volumen II características generales y educativas de los censos. Cuadro 19. Población de 10 y más años de edad en la República, por alfabetismo y sexo, según provincia, comarca indígena, distrito y corregimiento: censos 2023. 2023. https://www.inec.gob.pa/publicaciones/Default3.aspx?ID_PUBLICACION=1212&ID_CATEGORIA=19&ID_SUBCATEGORIA=71

[24] Instituto Nacional de Estadística y Censo. Volumen II características generales y educativas de los censos. Población de 4 y más años de edad en la República, por nivel de instrucción, según provincia, comarca indígena, sexo y grupos de edad: censos 2023. 2023. https://www.inec.gob.pa/publicaciones/Default3.aspx?ID_PUBLICACION=1212&ID_CATEGORIA=19&ID_SUBCATEGORIA=71

[25] Kish LWH. Survey Sampling. Biom Z. 1968;10(1):88–9.

[26] Cochran WG. Sampling techniques. 3 ed. New York: John Wiley & Sons. 1977.

[27] Mostafa SA, Ahmad IA. Recent developments in systematic sampling: A review. J Stat Theory Pract. 2018;12(2):290–310.

[28] Instituto Nacional de Estadística y Censo. Resultados Finales. 2010 [cited 2025 Feb 7]. https://www.inec.gob.pa/publicaciones/Default2.aspx?ID_CATEGORIA=13&ID_SUBCATEGORIA=59

[29] Vaissie P, Monge A, Husson F. Factoshiny: Perform Factorial Analysis from “FactoMineR” with a Shiny Application. 2024 [cited 2024 Nov 1]. https://cran.r-project.org/web/packages/Factoshiny/index.html

[30] Isaza DM, Restrepo BN, Arboleda M, Casas E, Hinestroza H, Yurgaqui T. Leishmaniasis: knowledge and practice in populations of the Pacific coast of Colombia. Rev Panam Salud Publica. 1999;6(3):177–84. doi: 10.1590/s1020-49891999000800005 10517095

[31] Patiño-Londoño SY, Salazar LM, Acero CT, Bernal IDV. Socio-epidemiological and cultural aspects of cutaneous leishmaniasis: conceptions, attitudes and practices in the populations of Tierralta and Valencia (Cordoba, Colombia). Salud Colect. 2017;13(1):123–38. doi: 10.18294/sc.2017.1079 28562730

[32] Hurtado LA, Calzada JE, Pineda V, González K, Santamaría AM, Cáceres L. Knowledge and risk factors related to Chagas’ disease in two Panamanian communities where Rhodnius pallescens is the main vector. Biomédica. 2014;34(2):260–70. doi: 10.1590/S0120-41572014000200012 24967931

[33] Alvar J, Yactayo S, Bern C. Leishmaniasis and poverty. Trends Parasitol. 2006;22(12):552–7. doi: 10.1016/j.pt.2006.09.004 17023215

[34] da Silva MAD, French O, Keck F, Skotnes-Brown J. Introduction: Disease Reservoirs: From Colonial Medicine to One Health. Med Anthropol. 2023;42(4):311–24. doi: 10.1080/01459740.2023.2214950 37522963 PMC12180427

[35] Keck F, Lynteris C. Zoonosis: prospects and challenges for medical anthropology. Med Anthropol Theory. 2018;5(3).

[36] Lynteris C. Introduction: infectious animals and epidemic blame. Fram Anim Epidemic Villains. 2019;1.

[37] Narat V, Alcayna-Stevens L, Rupp S, Giles-Vernick T. Rethinking Human-Nonhuman Primate Contact and Pathogenic Disease Spillover. Ecohealth. 2017;14(4):840–50. doi: 10.1007/s10393-017-1283-4 29150826

[38] King NB. Security, disease, commerce: ideologies of postcolonial global health. Soc Stud Sci. 2002;32(5–6):763–89.

[39] Costa-Neto E. The significance of the category “insect” for folk biological classification systems. J Ecol Anthropol. 2000;4(1).

[40] Morris B. Insects and human life. London: Routledge. 2004.

[41] Ulicsni V, Svanberg I, Molnár Z. Folk knowledge of invertebrates in Central Europe - folk taxonomy, nomenclature, medicinal and other uses, folklore, and nature conservation. J Ethnobiol Ethnomed. 2016;12(1):47. doi: 10.1186/s13002-016-0118-7 27729074 PMC5057442

[42] da Silva AF, Barão KR. Trends in the Brazilians’ engagement with insects on the internet. Insect Conserv Diversity. 2024;17(4):719–27. doi: 10.1111/icad.12740

[43] Leandro C, Jay-Robert P. Perceptions and representations of animal diversity: Where did the insects go? Biol Conserv. 2019;237:400–8.

[44] Ramos DL, Cunha WL, Evangelista J, Lira LA, Rocha MVC, Gomes PA. Ecosystem Services Provided by Insects in Brazil: What Do We Really Know? Neotrop Entomol. 2020;49(6):783–94. doi: 10.1007/s13744-020-00781-y 32462421

[45] Wallston KA, Wallston BS. Health locus of control scales. Research with the Locus of Control Construct. Elsevier. 2013. 189–243.

[46] Nichter M. Global health: why cultural perceptions, social representations, and biopolitics matter. University of Arizona Press. 2008.

[47] Davis A, Sharp J. Rethinking one health: emergent human, animal and environmental assemblages. Soc Sci Med. 2020;258:113093. doi: 10.1016/j.socscimed.2020.113093 32531688 PMC7369629

