# Supplementary material for: Putting behaviors into context for vector-borne diseases: Examining behaviors that may reduce exposure to disease vectors
Source: PLoS Negl Trop Dis. 2025 Aug 12;19(8):e0013365. doi: 10.1371/journal.pntd.0013365 (PMC12342304; doi:10.1371/journal.pntd.0013365)
Supplement: S1 Appendix — This appendix contains the complete version of the questionnaire used in the study. The original questionnaire was administered in Spanish. An English translation is provided for reference, following the original structure and wording as closely as possible. (DOCX) [file pntd.0013365.s001.docx]

Proyecto: **“**Determinantes sociales y ecológicos de las infecciones transmitidas por vectores de múltiples huéspedes en entornos rurales tropicales dinámicos”

**ENCUESTA DEL HOGAR (A)**

DEMOGRAFÍA

1. ¿Cuántas personas viven en su casa? ________________

[Indique el nombre, sexo, edad y educación de las personas que viven en la casa]

|  |  | Persona  entrevistada | Otras personas en la casa | | | | | |
| --- | --- | --- | --- | --- | --- | --- | --- | --- |
| ¿Mujer u hombre? | 0= mujer; 1=hombre |  |  |  |  |  |  |  |
| ¿Cuántos años tiene? | Años |  |  |  |  |  |  |  |
| ¿Hasta qué curso ha completado en la escuela? | 0=Ninguno, 1=Primaria incompleta; 2=Primaria completa, 3=Educación media, 4=Secundaria  5=Universitaria |  |  |  |  |  |  |  |

## CARACTERÍSTICAS DE LA VIVIENDA

1. ¿Dónde hace sus necesidades o como elimina las excretas?
   1. Servicio (letrina) de hueco
   2. Inodoro
   3. Río
   4. Otro _____________________
   5. No sabe
   6. No quiso responder

1. ¿Tiene depósitos de basura acumulada fuera de su casa o en el patio de la casa? _____ (Sí=1, No=0, 3=No sabe)
2. Tiene medidor de electricidad?
   1. Sí
   2. No
   3. No sabe

## RIQUEZA

1. Ahora vamos a hacerle algunas preguntas sobre la riqueza (bienes) de su hogar. ¿En su casa, hay…..? Si la respuesta es sí, indique la cantidad en la vivienda

|  | 1=Si, 0=No | ¿Cuántos en la casa en total? (#) |
| --- | --- | --- |
| Bicicleta |  |  |
| Motocicleta |  |  |
| Carro (auto) |  |  |
| Radio |  |  |
| Televisión |  |  |
| Televisión por Cable  (Sky, Claro, otros) |  |  |
| Internet (wifi, Claro, otros) |  |  |
| Teléfono fijo |  |  |
| Celular |  |  |
| Refrigerador |  |  |
| Lavadora |  |  |
| Máquina de coser |  |  |
| Escopeta o rifle |  |  |
| Computadora |  |  |
| Estufa (cocina) de gas |  |  |
| Ventilador/abanico |  |  |
| Generador de gas |  |  |
| Máquina de moler |  |  |
| Silla para caballos |  |  |
| Cama hecha de madera |  |  |

### LOS INSECTOS Y ANIMALES

1. ¿Utiliza mallas en las ventanas y puertas?
   1. Sí, en ambas (ventanas y puertas)
   2. Solamente ventanas
   3. Solamente puertas
   4. Algunas ventanas y/o puertas
   5. No
   6. No sabe
2. ¿Sufre molestias por insectos en su casa?
   1. Sí (pasar a: i-ii)
   2. No
      1. ¿Qué tipo de insectos?___________________________________________________________________
3. ¿Usted ha utilizado algún tratamiento para evitar los insectos en las paredes y pisos de su casa en los últimos 6 meses? (1=Sí, 2=No) (Anote el tipo de tratamiento p.e.j., Baygon, mechitas, velas, etc.) _____________________________________________________________
4. ¿Existen lugares donde molesten (pican) los insectos afuera de su casa?
   1. No
   2. Sí (pasar a: i y ii)
5. ¿Cuáles animales domésticos ustedes tienen en casa? ¿Cuántos? ¿Dónde duermen los animales?

| Animales | ¿Cuántos? (#) |
| --- | --- |
| Gatos |  |
| Gallinas |  |
| Cerdos |  |
| Patos |  |
| Conejos |  |
| Loros/Pericos |  |
| Caballos |  |
| Vacas |  |
| Perros |  |

SALUD

1. Prácticas

| ¿En general, uusted utiliza repelente, loción contra insectos, o “mechitas”? | Siempre | A veces | Nunca |
| --- | --- | --- | --- |

1. Percepción de la salud. Ahora, voy a leer algunas frases. Por favor diga si está muy en desacuerdo, en desacuerdo, neutro, de acuerdo, o muy de acuerdo.

|  | Muy en desacuerdo | En desacuerdo | Neutro | De acuerdo | Muy de acuerdo |
| --- | --- | --- | --- | --- | --- |
| Si me enfermo, es mi propio comportamiento (lo que hago) lo que ayuda a que me recupere pronto. |  |  |  |  |  |
| No importa lo que yo haga, si me voy a enfermar, me enfermaré. |  |  |  |  |  |
| El tener contacto regular con mi médico es la mejor manera de evitar enfermedad(es). |  |  |  |  |  |
| La mayoría de las cosas que afectan mi salud me ocurren por accidente. |  |  |  |  |  |
| Cuando no me siento bien, yo debería consultar a un  médico o profesional de salud. |  |  |  |  |  |
| Yo estoy en control total de mi salud. |  |  |  |  |  |
| Mi familia tiene mucho que ver con que yo me enferme o permanezca saludable. |  |  |  |  |  |
| Cuando me enfermo, es por algo que hice mal. |  |  |  |  |  |
| La suerte juega una gran parte en determinar qué tan pronto me recuperaré de una enfermedad |  |  |  |  |  |
| Los profesionales médicos controlan mi salud. |  |  |  |  |  |
| Es por mi buena suerte que tengo buena salud. |  |  |  |  |  |
| Lo principal que afecta mi salud, es lo que yo mismo hago. |  |  |  |  |  |
| Si yo me cuido, puedo evitar la(s) enfermedad(es). |  |  |  |  |  |
| Cuando me recupero de una enfermedad, es usualmente porque otras personas (por ejemplo: doctores/médicos, enfermeras, familia, amistades) me han estado cuidando bien. |  |  |  |  |  |
| No importa lo que haga, es probable que me enferme. |  |  |  |  |  |
| Si así es como debe ser, yo permaneceré saludable. |  |  |  |  |  |
| Si tomo las medidas correctas, yo puedo permanecer saludable. |  |  |  |  |  |
| Con respecto a mi salud, yo solo puedo hacer lo que mi médico me dice que haga. |  |  |  |  |  |
| Es por la gracia de Dios que cuento con buena salud. |  |  |  |  |  |
| Si llego a enfermarme, Dios me ayudará a recuperarme. |  |  |  |  |  |
| Si llego a enfermarme, el curandero me ayudará a recuperarme. |  |  |  |  |  |

### LEISHMANIASIS

1. ¿Ha oído o conoce qué es la Leishmaniasis? (Luego de responder “sí” o “no” mostrar la foto de una lesión sin dar detalles adicionales)
   1. Sí
   2. No (pasar a i y ii)
      1. ¿Cuál de estos nombres usted reconoce? (Marca todos)
         1. Grano malo
         2. Picada de Bejuco
         3. Picada de Bayano
         4. Ya te vi
         5. Lepra de la montaña
         6. Espundia
         7. Otro_____________
         8. Ninguno (Pasar a “Chagas”)
2. ¿Sabe usted cómo se transmite la Leishmaniasis? [Pregunte si la persona sabe cómo transmite y DESPUÉS pregunte a cada uno y marque todos con una respuesta positiva]
   1. Por la picadura de un bicho/insecto (pasar a 56)
   2. Por la picadura de un gusano
   3. Tocando un bejuco o una mata en el monte
   4. Se pega de otra persona que tiene la enfermedad
   5. Por falta de aseo/higiene personal
   6. Otro _______________
   7. No sabe
3. ¿Alguien en su casa tiene Leishmaniasis ahora mismo?

| Sexo | Edad | Lesión (notar dónde: cara, brazo, pierna, otro) | ¿Tomó medicina? 0=No; 1=Si, 3=No sabe. [Sí -- ¿Sabe que tipo de medicina? (anotar)] |
| --- | --- | --- | --- |
|  |  |  |  |
|  |  |  |  |
|  |  |  |  |

1. ¿Alguien en su casa ha tenido Leishmaniasis?

| Sexo | Edad (cuando tenía) | ¿Dónde vive? | Lesión (anotar dónde: cara, brazo, pierna, otro) | ¿Tomó medicina? 0=No; 1=Si, 3=No sabe. [Sí -- ¿Sabe qué tipo de medicina? (anotar)] |
| --- | --- | --- | --- | --- |
|  |  |  |  |  |
|  |  |  |  |  |
|  |  |  |  |  |

ENFERMEDAD DE CHAGAS

1. ¿Ha escuchado o conoce que es la enfermedad de Chagas?
   1. Sí (pasar a 63)
   2. No (pasar a: i y ii)
      1. ¿A cuál de estos reconoce? [Mostrar los chinches]
         1. Rhodnius pallescens
         2. Ninfas de Rhodnius pallescens
         3. Triatoma dimidiata
         4. Ninfas de Triatoma dimidiata
         5. Otro:______________________
         6. No sabe (pasar a fin de Chagas)
2. Vectores: Mostrar los chinches ¿A cuál de estos reconoce?
   1. Rhodnius pallescens
   2. Ninfas de Rhodnius pallescens
   3. Triatoma dimidiata
   4. Ninfas de Triatoma dimidiata
   5. Otro:______________________
3. ¿En el supuesto de que encuentre chinches, antes de tocarlos utiliza guantes o algún tipo de protección en las manos?
   1. Todo el tiempo
   2. A veces
   3. Nunca
   4. No sabe

**Project:** “Social and Ecological Determinants of Vector-Borne Infections with Multiple Hosts in Dynamic Tropical Rural Settings”

**HOUSEHOLD SURVEY (A)**

DEMOGRAPHICS

1. How many people live in your household?_______________

[Indicate the name, sex, age, and education level of each person living in the household

|  |  | Interviewed person | Other members of the household | | | | | |
| --- | --- | --- | --- | --- | --- | --- | --- | --- |
| Male or Female | 0= female; 1=male |  |  |  |  |  |  |  |
| Age | Age |  |  |  |  |  |  |  |
| Highest level of education completed | 0=None; 1=Incomplete Primary; 2=Completed Primary; 3=Middle School; 4=Secondary; 5=University |  |  |  |  |  |  |  |

## HOUSING CHARACTERISTICS

1. Where do you usually go to the bathroom or how do you dispose of human waste??

a. Pit latrine
b. Flush toilet
c. River
d. Other: _____________________
e. Don’t know
f. Did not answer

1. Do you have peridomestic debris accumulated around your house? _____ (Yes=1, No=0, 3=Don’t know)
2. Do you have an electric meter?
   1. Yes
   2. No
   3. Don’t know

## WEALTH

1. Now we’ll ask about the assets in your household. Do you have…? If yes, indicate the quantity in the home:

|  | 1=Si, 0=No | ¿Cuántos en la casa en total? (#) |
| --- | --- | --- |
| Bicicleta |  |  |
| Motorcycle |  |  |
| Car |  |  |
| Radio |  |  |
| Television |  |  |
| Cable TV (Sky, Claro, etc.) |  |  |
| Internet (Wi-Fi, Claro, etc.) |  |  |
| Landline phone |  |  |
| Mobile phone |  |  |
| Refrigerator |  |  |
| Washing machine |  |  |
| Sewing machine |  |  |
| Rfle |  |  |
| Computer |  |  |
| Gas stove |  |  |
| Fan |  |  |
| Gas generator |  |  |
| Grinding machine |  |  |
| Horse saddle |  |  |
| Wooden bed |  |  |

INSECTS AND ANIMALS

1. Do you have screens on your windows or doors?

a. Yes, on both (windows and doors)

b. Only windows

c. Only doors

d. Some windows and/or doors

e. No

f. Don’t know

1. Do you feel bothered by insects indoors?
   1. Yes (go to: i)
   2. No
      1. ¿What kind of insect?________________________________________________
2. Have you used any treatment to repel insects on your walls and floors in the last 6 months? (1=Yes, 2=No) (Note the type of treatment, e.g., Baygon, coils, candles, etc.) _____________________________________________________________
3. Do you feel bothered by insects outdoors?
   1. No
   2. Yes
4. What domestic animals do you have in your household? How many?

| Animals | How many? (#) |
| --- | --- |
| Chickens |  |
| Pigs |  |
| Ducks |  |
| Horses |  |
| Cows |  |

HEALTH

1. Practices

| Do you generally use insect repellent, lotion, or insect coils? | Always | Sometimes | Never |
| --- | --- | --- | --- |

**Health Perceptions**

1. Now I will read some statements. Please say if you strongly disagree, disagree, are neutral, agree, or strongly agree.

| **Statements** | **Strongly Disagree** | **Disagree** | **Neutral** | **Agree** | **Strongly Agree** |
| --- | --- | --- | --- | --- | --- |
| 1. If I become sick, it is my own behavior which determines how soon I will get well again. |  |  |  |  |  |
| 2. No matter what I do, if I am going to get sick, I will get sick. |  |  |  |  |  |
| 3. Having regular contact with my physician is the best way for me to avoid illness. |  |  |  |  |  |
| 4. Most things that affect my health happen to me by accident. |  |  |  |  |  |
| 5. Whenever I don't feel well, I should consult a medically trained professional. |  |  |  |  |  |
| 6. I am in control of my health. |  |  |  |  |  |
| 7. My family has a lot to do with my becoming sick or staying healthy. |  |  |  |  |  |
| 8. When I get sick, I am to blame. |  |  |  |  |  |
| 9. Luck plays a big part in determining how soon I will recover from an illness. |  |  |  |  |  |
| 10 Health professionals control my health. |  |  |  |  |  |
| 11. My good health is largely a matter of good fortune. |  |  |  |  |  |
| 12. The main thing which affects my health is what I myself do. |  |  |  |  |  |
| 13. If I take care of myself, I can avoid illness. |  |  |  |  |  |
| 14. Whenever I recover from an illness, it's usually because other people (for example, doctors, nurses, family, friends) have been taking good care of me. |  |  |  |  |  |
| 15. No matter what I do, I 'm likely to get sick. |  |  |  |  |  |
| 16. If it's meant to be, I will stay healthy. |  |  |  |  |  |
| 17. If I take the right actions, I can stay healthy. |  |  |  |  |  |
| 18. Regarding my health, I can only do what my doctor tells me to do. |  |  |  |  |  |
| 19. If I become sick, it is my own behavior which determines how soon I will get well again. |  |  |  |  |  |
| 20. No matter what I do, if I am going to get sick, I will get sick. |  |  |  |  |  |

LEISHMANIASIS

1. Have you heard about leishmaniasis?
   1. Yes
   2. No (go to a i)
      1. Have you heard any of these terms? (Mark all that apply)
         1. Grano malo
         2. Picada de Bejuco
         3. Picada de Bayano
         4. Ya te vi
         5. Lepra de la montaña
         6. Espundia
         7. Other____________
         8. None (Go to “Chagas”)
2. Do you know how Leishmaniasis is transmitted? [Ask open-ended first, then ask about each one and mark all that apply]

a. By the bite of a bug/insect (go to 56)
b. By the bite of a worm
c. By touching a bejuco or plant in the forest
d. Transmitted from another person who has the disease
e. Due to lack of personal hygiene
f. Other: _______________
g. Don’t know

1. Does anyone in your household currently have Leishmaniasis?

| Sex | Age | Lesion (where: face, arm, leg, other) | Took medication? 0=No; 1=Yes, 3=Don’t know. [If yes – What kind?] |
| --- | --- | --- | --- |
|  |  |  |  |
|  |  |  |  |
|  |  |  |  |

1. Has anyone in your household ever had leishmaniasis?

| Sex | Age (aat the time) | Where do they live? | Lesion (where: face, arm, leg, other) | Took medication? 0=No; 1=Yes, 3=Don’t know. [If yes – What kind?] |
| --- | --- | --- | --- | --- |
|  |  |  |  |  |
|  |  |  |  |  |
|  |  |  |  |  |

CHAGAS DISEASE

1. Have you heard of or do you know what Chagas disease is?
   1. Yes (go to 18)
   2. No (go to: i )
      1. Which of these do you recognize? [Show bugs]
         1. *Rhodnius pallescens*
         2. Nymphs of *Rhodnius pallescens*
         3. *Triatoma dimidiata*
         4. Nymphs of *Triatoma dimidiata*
         5. Other:______________________
         6. Don’t know (go to end of Chagas section)
2. Vectors: Show the bugs. Which of these do you recognize?
   1. *Rhodnius pallescens*
   2. Nymphs of *Rhodnius pallescens*
   3. *Triatoma dimidiata*
   4. Nymphs of *Triatoma dimidiata*
   5. Other:______________________
3. Would you use any kind of protection before having contact with kissing bugs?
   1. Always
   2. Sometimes
   3. Never
   4. Don’t know
