# Supplementary material for: Putting behaviors into context for vector-borne diseases: Examining behaviors that may reduce exposure to disease vectors
Source: PLoS Negl Trop Dis. 2025 Aug 12;19(8):e0013365. doi: 10.1371/journal.pntd.0013365 (PMC12342304; doi:10.1371/journal.pntd.0013365)
Supplement: S1 Table — Statements of internal, external powerful others, and external chance domains and scores ranging from strongly disagree to strongly agree. (DOCX) [file pntd.0013365.s002.docx]

**S1 Table**

| **Statements** | **Strongly Disagree**  **1** | **Disagree**  **2** | **Neutral**  **3** | **Agree**  **4** | **Strongly Agree**  **5** |
| --- | --- | --- | --- | --- | --- |
| 1. If I become sick, it is my own behavior which determines how soon I will get well again. ^2^ |  |  |  |  |  |
| 2. No matter what I do, if I am going to get sick, I will get sick. ^3^ |  |  |  |  |  |
| 3. Having regular contact with my physician is the best way for me to avoid illness.^1^ |  |  |  |  |  |
| 4. Most things that affect my health happen to me by accident. ^3^ |  |  |  |  |  |
| 5. Whenever I don't feel well, I should consult a medically trained professional.^1^ |  |  |  |  |  |
| 6. I am in control of my health. ^2^ |  |  |  |  |  |
| 7. My family has a lot to do with my becoming sick or staying healthy.^1^ |  |  |  |  |  |
| 8. When I get sick, I am to blame. ^2^ |  |  |  |  |  |
| 9. Luck plays a big part in determining how soon I will recover from an illness. ^3^ |  |  |  |  |  |
| 10 Health professionals control my health. ^1^ |  |  |  |  |  |
| 11. My good health is largely a matter of good fortune. ^3^ |  |  |  |  |  |
| 12. The main thing, which affects my health, is what I myself do. ^2^ |  |  |  |  |  |
| 13. If I take care of myself, I can avoid illness. ^2^ |  |  |  |  |  |
| 14. Whenever I recover from an illness, it's usually because other people (for example, doctors, nurses, family, friends) have been taking good care of me. ^1^ |  |  |  |  |  |
| 15. No matter what I do, I'm likely to get sick. ^3^ |  |  |  |  |  |
| 16. If it's meant to be, I will stay healthy. ^3^ |  |  |  |  |  |
| 17. If I take the right actions, I can stay healthy. ^2^ |  |  |  |  |  |
| 18. Regarding my health, I can only do what my doctor tells me to do. ^1^ |  |  |  |  |  |

^1^Powerful Others Locus of Control: statements 3, 5, 7, 10, 14, 18; ^2^Internal Locus of Control: statements 1, 6, 8, 12, 13, 17, ^3^Chance Locus of Control: statements 2, 4, 9, 11, 15, 16. Beside each locus of control statement is a scale that ranges from strongly disagree (1) to strongly agree (5). For each statement, the interviewer will circle the number that represents the extent to which the respondent agrees or disagrees with that statement, allowing a range score of 6-30 per Locus of Control.
