# Supplementary material for: Putting behaviors into context for vector-borne diseases: Examining behaviors that may reduce exposure to disease vectors
Source: PLoS Negl Trop Dis. 2025 Aug 12;19(8):e0013365. doi: 10.1371/journal.pntd.0013365 (PMC12342304; doi:10.1371/journal.pntd.0013365)
Supplement: S3 Table — Odds ratios (OR), 95% confidence intervals (CI), and p-values are shown for each predictor variable across the three outcomes. For binary predictors, the odds ratio represents the odds of the outcome for the ‘Yes’ or ‘Male’ category, compared to the ‘No’ or ‘Female’ (reference) category. (DOCX) [file pntd.0013365.s004.docx]

**S3 Table**

|  | **Do you have screens on your windows/doors?** | | | **Have you used any treatment to prevent insects on the walls and floors of your home in the last 6 months?** | | | **Do you have debris accumulated around your house?** | | |
| --- | --- | --- | --- | --- | --- | --- | --- | --- | --- |
| **Predictors** | **OR** | **95% CI** | **p-value** | **OR** | **95% CI** | **p-value** | **OR** | **95% CI** | **p-value** |
| **Sex** |  |  |  |  |  |  |  |  |  |
| Female | — | — |  | — | — |  | — | — |  |
| Male | 0.94 | 0.49, 1.82 | 0.90 | 1.08 | 0.59, 2.00 | 0.80 | 1.86 | 0.97, 3.61 | 0.07 |
| **Consumer goods and appliances** | 1.44 | 1.23, 1.71 | <0.01 | 0.94 | 0.81, 1.08 | 0.40 | 1.02 | 0.88, 1.19 | 0.80 |
| **Agricultural wealth** | 0.92 | 0.69, 1.17 | 0.50 | 0.91 | 0.74, 1.12 | 0.40 | 0.97 | 0.75, 1.20 | 0.80 |
| **Had heard about ACL or any other term** |  |  |  |  |  |  |  |  |  |
| No | — | — |  | — | — |  | — | — |  |
| Yes | 0.84 | 0.29, 2.44 | 0.80 | 2.65 | 0.94, 7.92 | 0.07 | 0.88 | 0.29, 2.77 | 0.80 |
| **Understood ACL is transmitted by an insect** ^a^ |  |  |  |  |  |  |  |  |  |
| No | — | — |  | — | — |  | — | — |  |
| Yes | 1.26 | 0.60, 2.66 | 0.50 | 1.58 | 0.81, 3.11 | 0.20 | 0.99 | 0.48, 2.05 | >0.90 |
| **Had a case of ACL in the household** |  |  |  |  |  |  |  |  |  |
| No/Does not know | — | — |  | — | — |  | — | — |  |
| Yes | 0.84 | 0.41, 1.71 | 0.60 | 0.77 | 0.40, 1.47 | 0.40 | 2.33 | 1.17, 4.75 | 0.02 |
| **Had heard about CD** |  |  |  |  |  |  |  |  |  |
| No | — | — |  | — | — |  | — | — |  |
| Yes | 1.86 | 0.67, 5.21 | 0.20 | 0.84 | 0.33, 2.20 | 0.70 | 1.34 | 0.48, 3.66 | 0.60 |
| **Have seen kissing bugs** |  |  |  |  |  |  |  |  |  |
| No | — | — |  | — | — |  | — | — |  |
| Yes | 1.23 | 0.60, 2.57 | 0.60 | 0.78 | 0.39, 1.55 | 0.50 | 0.73 | 0.36, 1.50 | 0.40 |
| **Bothered by insects indoors** |  |  |  |  |  |  |  |  |  |
| No | — | — |  | — | — |  | — | — |  |
| Yes | 0.78 | 0.38, 1.59 | 0.50 | 0.96 | 0.49, 1.89 | >0.90 | 0.42 | 0.20, 0.84 | 0.02 |
| **Bothered by insects outdoors** |  |  |  |  |  |  |  |  |  |
| No | — | — |  | — | — |  | — | — |  |
| Yes | 1.29 | 0.58, 2.94 | 0.50 | 1.06 | 0.50, 2.23 | 0.90 | 0.843 | 0.39, 1.80 | 0.60 |
| **Powerful Others** | 0.96 | 0.86, 1.08 | >0.90 | 0.96 | 0.86, 1.06 | 0.40 | 1.02 | 0.91, 1.14 | 0.70 |
| **Internal** | 1.08 | 0.95, 1.24 | 0.20 | 1.07 | 0.95, 1.21 | 0.30 | 1.04 | 0.92, 1.18 | 0.50 |
| **Chance** | 1 | 0.91, 1.09 | >0.90 | 0.95 | 0.87, 1.03 | 0.20 | 0.89 | 0.82, 0.98 | 0.01 |

OR: Odds Ratio; 95% CI: 95% Confidence Interval; ACL: American Cutaneous Leishmaniasis; CD: Chagas disease.

^a^ Respondents were asked “Do you know how leishmaniasis is transmitted?” and were provided with a list of options to choose from. Those who answered, “It is transmitted by the bite of an insect” were considered to understand the correct mode of transmission and were coded as "yes" (1), while all other incorrect options selected by the respondents were coded as "no" (0), indicating a lack of understanding.
